# The Inconsistent Nature of Heart Rate Variability During Sleep in Normal Children and Adolescents

**DOI:** 10.3389/fcvm.2020.00019

**Published:** 2020-02-21

**Authors:** Anna Kontos, Mathias Baumert, Kurt Lushington, Declan Kennedy, Mark Kohler, Diana Cicua-Navarro, Yvonne Pamula, James Martin

**Affiliations:** ^1^Department of Respiratory and Sleep Medicine, Women's and Children's Hospital, Adelaide, SA, Australia; ^2^School of Paediatrics and Reproductive Health, Robinson's Research Institute, University of Adelaide, Adelaide, SA, Australia; ^3^School of Electrical and Electronic Engineering, University of Adelaide, Adelaide, SA, Australia; ^4^School of Psychology, Social Work and Social Policy, University of South Australia, Adelaide, SA, Australia; ^5^School of Psychology, University of Adelaide, Adelaide, SA, Australia

**Keywords:** heart rate variability, children, sleep, circadian rhythm, ultradian rhythm

## Abstract

**Introduction:** Cardiac function is modulated by multiple factors including exogenous (circadian rhythm) and endogenous (ultradian 90–110 min sleep cycle) factors. By evaluating heart rate variability (HRV) during sleep, we will better understand their influence on cardiac activity. The aim of this study was to evaluate HRV in the dark phase of the circadian rhythm during sleep in healthy children and adolescents.

**Methods:** One 3 min segment of pre-sleep electrocardiography (EEG) and 3, 6 min segments of electrocardiography recorded during polysomnography from 75 healthy children and adolescents were sampled during progressive cycles of slow wave sleep (SWS1, SWS2, SWS3). Three, 3 min segments of rapid eye movement sleep (REM) were also assessed, with REM1 marked at the last REM period before awakening. Studies that recorded REM3 prior to SWS3 were used for assessment. HRV variables include the following time domain values: mean NN (average RR intervals over given time), SDNN (Standard Deviation of RR intervals), and RMSSD (root Mean Square of beat-to-beat Differences). Frequency domain values include: low frequency (LF), high frequency (HF), and LF:HF.

**Results:** Mixed linear effects model analysis revealed a significant difference in time and frequency domain values between sleep cycles and stages. Mean NN was lowest (highest heart rate) during pre—sleep then significantly increased across SWS1-3. Mean NN in SWS1 was similar to all REM periods which was significantly lower than both SWS2 and SWS3. SDNN remained at pre-sleep levels until SWS3, and then significantly increased in REM1&2. There was a large drop in LF from pre-sleep to SWS1. As cycles progressed through the night, LF remains lower than awake but increases to awake like levels by REM2. RMSSD and HF were lowest in pre-sleep and increased significantly by SWS1 and remain high and stable across stages and cycles except during the REM3 period where RMSSD decreased.

**Conclusion:** Our results demonstrate that there are considerable changes in the spectral analysis of cardiac function occurring during different sleep stages and between sleep cycles across the night. Hence, time of night and sleep stage need to be considered when reporting any HRV differences.

## Introduction

Cardiac function and regulation is complex and involves multiple factors that influence the timing (chronotropy) and contractile force (ionotropy) of cardiac muscle tissue (1). Broadly the factors can be divided into those which are (1) organ specific (the composition–muscle fiber/connective tissue) and basal metabolic state (cell size/connectivity) of the cardiac muscle tissue) and (2) those which operate on the cardiovascular system (e.g., exogenous factors such as light exposure and ambient temperature and endogenous factors such as central pattern generators) and activate afferent pathways or influence central neural networks and reflex circuits needed to maintain metabolic functioning, homeostasis and permit adaptive behaviors such as digestion and sleep (1–3). These latter influences on cardiac function can be assessed using heart rate variability, a commonly used mathematical manipulation of the electrocardiogram signal.

Both linear and non-linear techniques have been developed to determine HRV parameters such as variance in the cardiac timing including the average RR intervals over given time (mean NN), Standard Deviation of RR intervals (SDNN), and root Mean Square of beat-to-beat Differences (rMSSD). Variance in the power distribution across the frequency range is also calculated using Fast Fourier transforms to determine changes in low frequency (LF, 0.15–0.40 Hz) and high frequency (HF, 0.03–0.15 Hz) heart beat regularity. These variables have been proposed to be indicators of the relative tone of the two arms of the autonomic nervous system, the parasympathetic (HF) and sympathetic nervous systems (LF) and hence sympathovagal activity (LF:HF) acting directly on the cardiac tissue (3–5).

More recent evaluation of the frequency domain parameters suggests that all frequency domain variables (LF, HF, and LF:HF) are more likely to represent parasympathetic drive alone (6) especially LF in the supine position during paced breathing (7). It has also been postulated that LF and oscillations in the 0.1 Hz range represent vagal control of the baroreflex (8). In support of this hypothesis are studies which show that manipulation of the baroreflex response alone demonstrates a positive correlation in LF power while manipulation of the sympathetic innervation to the cardiac tissue does not alter LF power (6, 8).

Sleep is a time where there is considerable change in the cardiac function and in vascular tone of cutaneous vessels and thermoregulation (9, 10). Hence by investigating HRV during sleep where we know there are dynamic changes occurring both in cardiac function and vascular compliance across the night, we may better understand the genesis of the different HRV parameters in the ECG signal.

Occurring in the dark phase of the circadian cycle the propensity to sleep increases in humans after core body temperature reaches its peak (acrophase ~2,100 h) and then body temperature begins to fall (11, 12). Upon sleep onset, sympathetic tone to the musculature decreases while cardiac parasympathetic activity/vagal tone increase and heart rate decreases (13). This is paralleled by a drop in blood pressure and an increase in peripheral vasculature dilatation to cutaneous vessels to dissipate heat (11, 14). As sleep progresses, and core body temperature reaches its lowest point across the dark phase (nadir 0300–0500), the processes reverse and sleep propensity decreases, and body temperature and heart rate then begin to rise before wake (9). This latter change is coupled with a reduction in peripheral vascular compliance which is proposed to occur in order to increase core and brain temperature to facilitate arousal and awakening. Hence vascular compliance plays a large role in sleep propensity and the dark phase of the circadian cycle. Whether LF (as a potential marker of baroreflex/vascular compliance) also follows the change in peripheral vascular compliance and whether this trend is also evident in vagal measurements involved in cardiac timing (mean NN, SDNN, and RMSSD) and HF at the same time has not been addressed in the literature.

Superimposed on the dark phase of the circadian cycle is a 90–110 min ultradian rhythm consisting of two distinct sleep stages [non-rapid eye movement sleep (NREM) and rapid eye movement sleep (REM)] (15). Heart rate also demonstrates characteristic ultradian rhythmic changes with slower heart rate in NREM (16), especially in NREM3/4—known as slow wave sleep (SWS) and higher rates in REM (17). Although the mechanisms governing the control of ultradian rhythmicity are not fully understood, it is well-accepted that the autonomic nervous system and its control on both the cardiac tissue and peripheral vasculature are major contributors to the ultradian changes in cardiac function observed during sleep (18).

By investigating the changes in HRV variables in stable periods of sleep across the night where we know that there is a circadian change in body temperature and where the largest difference in frequency and amplitude in the EEG signals are reported (REM and SWS) and hence evidence of the peak and trough of each individual sleep cycle across the night are evident, we may better understand the relationship of HRV variables and the physiology of sleep. This study therefore aims to examine the effects of time-of-night and sleep stage (SWS vs. REM) on HRV parameters in a cohort of healthy children and adolescents.

## Methods

### Subjects

The polysomnographic (PSG) data of 118 children aged 3.1–15.1 years, who had previously participated, as healthy control children from the community, in three sleep projects, through the Women's and Children's Hospital, Sleep Disorders Unit were analyzed in this study (19–21). Parental questionnaires of the child's health and the results of the PSG confirmed their status as non-snoring, healthy children. Height was measured using a wall mounted stadiometer and weight was measured using a digital scale with a resolution of 0.1 kg. Body mass index, percentile and exact age at testing were calculated using a website algorithm (http://www.bcm.edu/cnrc/bodycomp/bmiz2.html).

### Polysomnography

The Compumedics Sleep Systems (Melbourne, Australia) was used to collect electroencephalograhic (EEG), left and right electro-oculographic (EOG), sub-mental and diaphragmatic electromyographic (EMG) data. Leg movement was assessed by electromyography, heart rate by electrocardiogram (ECG), oro-nasal airflow by thermistor and nasal pressure, respiratory movements of the chest and abdominal wall using uncalibrated respiratory inductive plethysmography (RIP) and arterial oxygen saturation (SaO_2_) by pulse oximetry (Nellcor N-595; 2 s averaging time). Children were continuously monitored via infrared camera by a pediatric sleep technician who also documented observations of sleep behavior, which included the presence or absence of snoring. All sleep studies were conducted in the same environment at the same ambient temperature throughout sleep.

### Electrocardiographic Recordings

The overnight ECG signals which were sampled at a rate of 512 Hz, were used to determine HRV values.

### Stage Choice and Segment Length

Pre-sleep HRV values were derived from a single segment of ECG data taken 3 min just prior to sleep onset. Segments of SWS and REM sleep were chosen for analysis because of the reported relative difference in stability of the EEG, ECG signals and respiratory rate. Sleep stages are not homogenously distributed throughout sleep, with more SWS in the beginning of sleep and REM more prevalent in the later hours, closer to awaking (9). Also, the time spent in each stage is significantly different with longer time spent in SWS compared to REM sleep. To ascertain the HRV in the most vegetative state (no movement artifact) and to maximize the number of recordings that could be considered we chose three clear consecutive segments from sleep onset of 6 min lengths each to analyze during SWS and three consecutive segment taken of 3 min lengths each during REM sleep. Although long term HRV measurements (e.g., 24 h Holter recordings) are considered predicative of pathology (22) the aim of this study was to look at the most stable cardiac signal, independent of the influence that other factors can contribute to changes in the cardiac signal such as exercise/movement, respiratory rate, ambient temperature fluctuations, food/drink consumption and changes in physical exertion. HRV parameters we have chosen to investigate can be evaluated using both short and ultra-short measurements (1 × 3 min Pre-sleep, 3 × 6 min of SWS, and 3 × 3 min REM) (22) especially if the participant is stationary and there is little variability in heart rate over the segment (23). Stage 2 NREM was not analyzed as the processes that drive Stage 2 ascending into Stage 3/4 and Stage 2 descending from a period of Stage 3/4 are unlikely to be the same and may confound the interpretation of the results.

**Figure 1:**
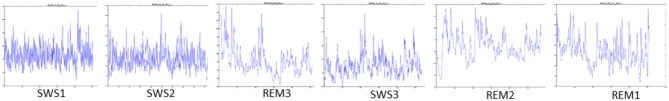
Tachnograms in temporal order from a male participant (8.25 y, BMI 17.6), during SWS1, SWS2, REM3, SWS3, REM2, and REM1.

Each segment was identified with a number corresponding to its timing in the sleep cycle, e.g., SWS1 (first SWS, early sleep), SWS2 (second SWS) etc. REM segments were labeled in reverse with the last REM stage (closest to morning wake time) recorded as REM1, REM2, and REM3 (Figure 1). The latter occurred closer to the middle of total sleep. So that we captured SWS and REM stages in a consistent pattern across the night we only used recording where REM3 preceded SWS3 for analysis. Therefore data included were taken as follows: SWS1, SWS2, REM3, SWS3, REM2, REM1.

When a corresponding sleep stage did not have a clear sequence of uninterrupted sleep containing no movement or respiratory events (apnea, hyponea, and snoring), then no data was recorded for that cycle, e.g., some participants only have recordings for SWS1 and SWS2 but not SWS3, etc.

### Heart Rate Variability Analysis

For HRV analysis, QRS locations were automatically detected in the ECG channel of PSG using Alfonso's filterbank algorithm (24). Tachograms of RR time series were visually scanned for artifacts. Segments with artifacts were excluded from analysis. The HRV variables were computed in accordance with Task Force recommendations using MatLab (25). Frequency domain analysis was conducted on RR time series interpolated at 250 ms using the Fast Fourier Transform. We examined the low frequency component of the spectrum analysis (0.03–0.15 Hz), which is reported to correspond to the baroreflex control of blood pressure/vascular compliance. The primary effect of the baroreflex is on total peripheral resistance via the rostral ventral lateral medulla activation or inhibition of sympathetic vasoconstrictor neurons (26). We also evaluated the high frequency (HF) component of the spectrum analysis (0.15–0.40 Hz), which is reported to correspond to the vagal control of heart rate and the oscillations induced by respiratory activity and is, therefore, a marker of parasympathetic activity (22). Spectral bands were chosen according to Task Force standards. It is possible that some of the HF power may have been excluded in very small children due to higher breathing rates (>0.4 Hz). Time domain analyses of HRV were also undertaken with the root Mean Square of the Successive Differences between neighboring RR intervals (rMSSD) thought to be indicative of vagal mediated changes in HRV and correlates with HF but less effected by respiratory changes and standard deviation of the inter-beat interval of normal sinus beats (SDNN) indicative of both sympathetic and parasympathetic activity, SDNN correlates with LF. Short term recording in resting conditions reflect parasympathetic mediated respiratory sinus arrhythmia (RSA) (22).

### Data Analysis

To corroborate parental report of normal sleep in the cohort, the PSG results were used to objectively determine that children did not have an underlying sleep condition (e.g., sleep disordered breathing). An experienced sleep technician scored the studies according to standardized sleep stage protocol by Kales and Rechtschaffen (27) and pediatric ventilatory criteria set by the American Association of Sleep Medicine (28). A mixed linear effects model analysis was performed to account for repeated measurements for HRV variables and cycles (SWS1-3 and REM1-3, fixed effects) and pairwise comparisons using the fixed effect model were used to determine within subject variability between pre-sleep and sleep cycles (7 levels) using SPSS, version 24. Unstructured repeated covariance type was used. Age, BMIz score and gender were correlated with each individual HRV variable (average of 7 values presleep, SWS1-3, REM1-3) to determine if there was an association between variable that would need to be considered as covariates in the mixed-model analysis. *Post-hoc* comparisons using Bonferroni Correction were performed to determine within subject differences between sleep cycles with a *p* < 0.05 considered significant.

## Results

### Anthropometric and Sleep Values

Of the initial 118 children, the REM3 period preceded the SWS3 period in 75 children. Hence the data present is from those children (50 females and 25 males) as SWS3 preceded REM3 in 42 recordings. Preliminary analyses indicted no significant gender differences in any anthropometric and sleep value. Anthropometric and sleep cycles onset HRV values are reported in Table 1.

**Table 1:** Minimum, maximum and mean (SD) anthropometric and sleep values (75 children).

**Anthropometrics**	**Mean (SD)**	**Minimum**	**Maximum**
Age (y)	8.5 (2.6)	3.1	13.2
Height (cm)	130.3 (16.3)	89.0	192.0
Weight (kg)	30.8 (11.7)	13.0	82.7
BMI (age and gender adjusted)	17.6 (2.6)	13.4	26.8
BMI z-score	0.4 (0.8)	–1.6	2.2
**Sleep^a^**			
Sleep onset time (h:min)	21:40 (0:42)	20:01	23:53
SWS1 onset time (h:min)	22:58 (1:08)	21:07	23:51
SWS2 onset time (h:min)	24:44 (1:31)	21:10	02:47
REM3 onset time (h:min)	01:39 (1:13)	23:09	03:31
SWS3 onset time (h:min)	02:40 (1:39)	21:51	05:21
REM2 onset time (h:min)	03:26 (1:23)	23:14	05:54
REM1 onset time (h:min)	05:01 (1:17)	01:15	07.52

a *SWS, slow wave sleep. SWS cycles are labeled relative to initial sleep onset: SWS cycle after sleep onset = 1st SWS, SWS cycle after sleep onset but one = 2nd SWS and SWS cycle after sleep onset but two = 3rd SWS cycle. REM, rapid eye movement sleep. REM cycles are labeled relative to sleep offset: REM cycle prior to sleep offset = REM1, REM cycle preceding sleep offset but one = REM2 and REM cycle preceding sleep offset but two = REM3 (see Figure 1)*.

### Heart Rate Variability: Sleep Stage and Sleep Cycle

To determine if any of our variables are related to age, gender or BMIz, we conducted Pearson correlations between these variables and averaged HRV variables. There were no correlations between our averaged variables and the age, gender or BMIz and hence covariance was not required for analysis. Mean and standard error in HRV values are provided in Table 2. Mean differences and standard error in HRV values are in **Table 4**.

**Table 2:** Mean (SD) heart rate variability values according to sleep stages and sleep cycle.

**Cycle onset time**	**Sleep stage and sleep cycle**	**Heart rate variability**					
		**Time domain variables**			**Power domain variables**		
		**Mean NN**	**SDNN**	**rMSSD**	**LF_FFT**	**HF_FFT**	**LF:HF**
21:40	Pre-sleep	704.6 (9.4)	55.9 (3.7)	48.1 (4.6)	1440.8 (388.0)	944.6 (139.6)	1.83 (0.24)
22:58	SWS1	775.7 (10.3)	56.1 (4.1)	68.8 (5.5)	639.4 (94.7)	1768.5 (294.9)	0.68 (0.09)
24:44	SWS2	809.4 (11.5)	57.9 (4.0)	70.8 (5.9)	750.3 (91.5)	1974.8 (346.0)	0.69 (0.07)
01:39	REM3	754.7 (11.1)	62.6 (4.3)	61.8 (5.9)	937.3 (170.8)	1483.1 (364.5)	1.16 (0.12)
02:40	SWS3	824.6 (13.0)	59.7 (4.5)	71.5 (6.7)	922.3 (148.9)	2059.8 (370.1)	1.06 (0.20)
03:26	REM2	772.9 (11.2)	71.6 (5.2)	74.0 (7.3)	1501.2 (243.2)	2299.4 (264.6)	1.82 (0.30)
05:01	REM1	763.7 (10.4)	68.2 (4.3)	67.1 (5.9)	1316.7 (235.4)	2020.5 (377.1)	1.91 (0.26)

*Note: Although Stage 3 and 4 are combined for reporting purposes, we restricted our SWS analysis to Stage 4 sleep (Standard Deviation)*.

*SWS, slow wave sleep. SWS cycles are labeled relative to sleep onset: SWS cycle after sleep onset = 1st SWS, SWS cycle after sleep onset but one = 2nd SWS and SWS cycle after sleep onset but two = 3rd SWS cycle. REM, rapid eye movement sleep. REM cycles are labeled relative to sleep offset: REM cycle prior to sleep offset = REM1, REM cycle preceding sleep offset but one = REM2 and REM cycle preceding sleep offset but two = REM3 (see Figure 1)*.

#### Time Domain Variables

**Figure 2:**
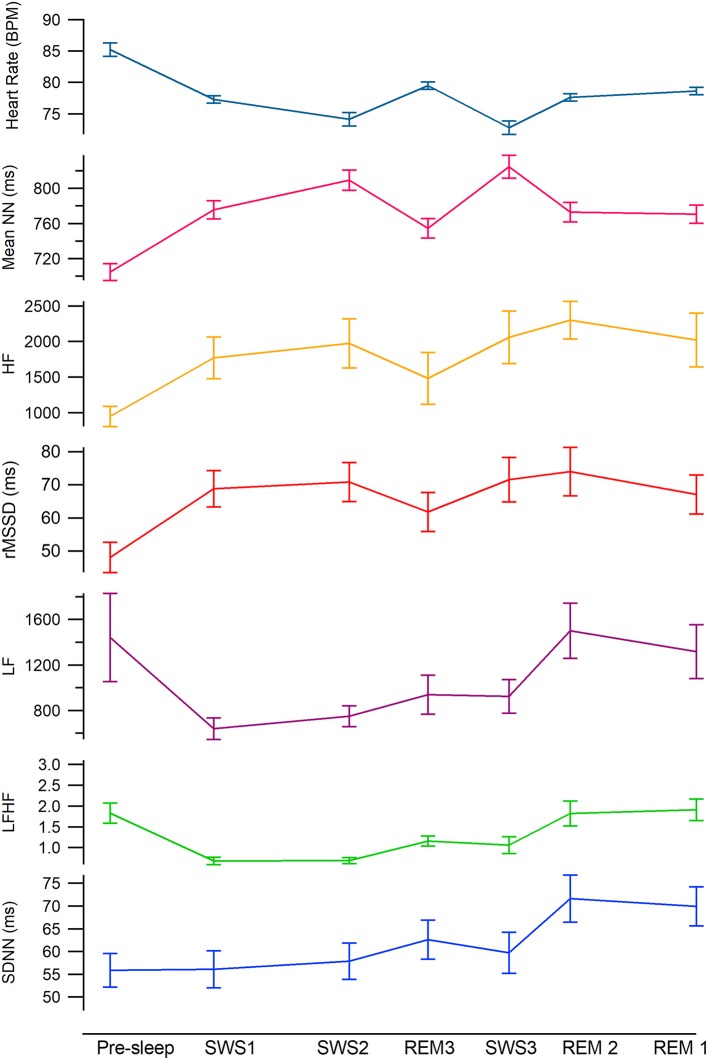
Mean heart rate variability values across the night by sleep stage. From the top teal: heart rate, hot pink: meanNN, gold: HF, red: rMSSD, magenta: LF, green: LF:HF, blue: SDNN. Mean time of each epoch are as follows; Sleep onset, 2140 SWS1, 2258, SWS2, 2444, REM3, 0139, SWS3, 0240, REM2, 0326, REM1, 0501 (see Table 1 for time standard deviations).

The test for fixed effects, revealed a significant main effect for sleep cycle (Table 3). **Mean NN**, was significantly lower in pre-sleep compared to all other sleep cycle/stages. While asleep mean NN in SWS1 and all REM periods were similar and significantly lower (higher heart rate) compared to SWS2 and SWS3 (Tables 2–4). **SDNN** was similar in pre-sleep and all SWS cycles and REM3 but increased significantly in REM1 and REM2 (Tables 2–4). **RMSSD** was also lowest in pre-sleep compared to all cycles and stages (Tables 2–4). Apart from a drop in REM3, RMSSD was stable across the night regardless of sleep stage (see Figure 2).

**Table 3:** Fixed effects values for sleep cycle.

**Heart rate variability variable**	**Sleep cycle**
Mean NN	*F*_(6,60.5)_ = 40.8, *p* < 0.001
SDNN	*F*_(6,56.3)_ = 3.7, *p* < 0.004
rMSSD	*F*_(6,59.5)_ = 3.9, *p* < 0.003
LF	*F*_(6,41.6)_ = 6.7, *p* < 0.001
HF	*F*_(6,58.0)_ = 3.2, *p* < 0.01
LF:HF	*F*_(6,55.6)_ = 9.5, *p* < 0.001

**Table 4:** Mean (Standard Error) differences between heart rate variability values according to sleep stage and sleep cycle.

**Sleep stage and sleep cycle**	**Heart rate variability**					
	**Time domain variables**			**Power domain variables**		
	**Mean NN**	**SDNN**	**rMSSD**	**LF**	**HF**	**LF:HF**
Pre-sleep SWS1	−73.0^***^ **(6.7)**	–0.3 (4.0)	−20.7^*^ **(5.5)**	515.1^**^ **(169.0)**	−823.9^*^ **(269.9)**	1.14^***^ **(0.25)**
Pre-sleep SWS2	−106.6^***^ **(8.8)**	–2.0 (4.0)	−22.7^*^ **(5.5)**	429.2^*^ **(178.2)**	−1030.2^*^ **(312.3)**	1.14^***^ **(0.25)**
Pre-sleep REM3	−50.9^***^ **(9.9)**	–6.7 (4.5)	−13.7^*^ **(5.2)**	–217.2 (234.4)	−538.5^*^ **(212.5)**	0.67^*^ **(0.24)**
Pre-sleep SWS3	−125.0^***^ **(10.6)**	–3.8 (4.6)	−23.4^*^ **(6.3)**	232.2 (235.4)	−1115.3^*^ **(328.6)**	0.77^**^ **(0.28)**
Pre-sleep REM2	−69.3^***^ **(9.7)**	**−15.7** (4.5)^*^	−25.0^**^ **(6.1)**	–346.7 (280.8)	−1354.8^*^ **(417.5)**	0.004 (0.34)
Pre-sleep REM1	−60.1^***^ **(10.0)**	–12.3 (4.3)	−19.0^*^ **(5.6)**	–217.2 (2345)	−1254.5^*^ **(355.9)**	0.08 (0.34)
**SWS1** vs. SWS2	**−33.8** (5.4)^***^	–1.48 (2.7)	–2.0 (3.8)	–85.9 (110.4)	–206.4 (210.4)	0.02 (0.7)
SWS1 vs. SWS3	**−49.0** (7.4)^***^	–4.0 (3.7)	–2.7 (5.1)	–282.9 (164.1)	–291.3 (269.1)	−038^*^ **(0.17)**
SWS2 vs. SWS3	−15.2^*^ **(7.4)**	–2.6 (2.3)	–0.7 (3.4)	–196.9 (139.3)	–84.9 (144.1)	−0.37^*^ **(0.17)**
SWS1 vs. REM3	20.9^*^ **(8.0)**	−6.4^*^ **(3.1)**	7.0 (4.3)	−297.9^*^ **(139.1)**	285.3 (234.5)	−0.48^***^ **(0.10)**
SWS1 vs. REM2	2.7 (8.9)	−15.5^**^ **(3.9)**	–5.1 (56)	−847.4^***^ **(228.6)**	–530.9 (415.5)	−1.14^***^ **(0.27)**
SWS1 v REM1	11.9 (9.3)	−12.1^*^ **(3.5)**	1.7 (5.2)	−677.3^*^ **(229.7)**	–252.1 (301.4)	−1.22^***^ **(0.25)**
**SWS2** v REM3	54.8^***^ **(5.9)**	–4.6 (3.3)	**9.0** (4.1)^*^	–211.9 (174.5)	–85.0 (144.2)	−0.48^***^ **(0.11)**
SWS2 v REM2	36.5^***^ **(7.5)**	−13.7^***^ **(3.7)**	–3.1 (4.8)	−775.9^*^ **(221.3)**	–324.5 (379.9)	−1.14^***^ **(0.26)**
SWS2 v REM1	45.7^***^ **(9.1)**	−10.3^**^ **(3.2)**	3.7 (4.4)	−591.4^*^ **(234.5)**	–45.7 (318.8)	−1.22^***^ **(0.23)**
**SWS3** v REM3	69.9^***^ **(8.6)**	–2.9 (3.9)	9.7^*^ **(4.6)**	15.0 (205.2)	576.7 (289.6)	–0.10 (0.19)
SWS3 v REM2	51.7^***^ **(8.3)**	−11.9^**^ **(3.9)**	–2.4 (4.9)	−578.9^*^ **(245.4)**	–239.6 (321.7)	−0.76^*^ **(0.26)**
SWS3 v REM1	60.9^***^ **(10.8)**	−8.5^*^ **(3.5)**	4.3 (4.5)	–394.5 (256.7)	–39.2 (304.8)	−0.84^**^ **(0.24)**
**REM3** vs. REM2	−198.2^*^ **(7.7)**	−9.1^*^ **(4.0**)	−12.2^*^ **(5.1)**	–563.9 (294.6)	−816.3^*^ **(356.5)**	−0.66^**^ **(0.25)**
REM3 vs. REM1	–9.0 (8.5)	–5.6 (2.8)	–5.3 (3.4)	–379.4 (284.9)	−537.4^*^ **(259.9)**	−0.75^**^ **(0.22)**
**REM2** vs. REM1	–9.2 (7.5)	–3.4 (3.1)	–6.8 (4.2)	184.5 (141.9)	278.9 (379.2)	0.09 (0.18)

* *Denotes p < 0.05*,

** *p < 0.01*,

and

*** *p < 0.001 (significant differences are bolded)*.

*SWS, slow wave sleep. SWS cycles are labeled relative to initial sleep onset: SWS cycle after sleep onset = 1st SWS, SWS cycle after sleep onset but one = 2nd SWS and SWS cycle after sleep onset but two = 3rd SWS cycle. REM = rapid eye movement sleep. REM cycles are labeled relative to sleep offset: REM cycle prior to sleep offset = REM1, REM cycle preceding sleep offset but one = REM2 and REM cycle preceding sleep offset but two = REM3 (see Figure 1)*.

#### Power Domain Variables

The test for fixed effects revealed there was a significant difference between cycles (Table 3). **LF** significantly dropped by half from pre-sleep to SWS1 and remained low as the night progressed regardless of stage (SWS2, SWS3, and REM3 not significantly different). The largest increase occurred in REM2 (see Table 4) returning to pre-sleep like values by REM1. **HF** was lowest during pre-sleep and doubled by SWS1 and continued to remain higher regardless of stage or time of night. **LF:HF** significantly reduced from pre-sleep to SWS1 and similar to LF continued to increase as the night progressed independent of sleep stage.

## Discussion

The present findings indicate that HRV parameters in healthy children and adolescents vary throughout the sleep/wake cycle and between sleep stages. Children/adolescents can have the same heart rate and very different timing and power HRV variables, depending where the cycle is positioned across sleep. HRV values can be similar or higher than pre-sleep levels in REM sleep, yet heart rate is not the same or higher. These differences may arise from modifying factors that are part of the patterned effect that drive sleep propensity and sleep behavior. Furthermore, the outcome of the mixed model analysis supports that this phenomenon in HRV is independent of age in children and adolescents aged 3–13 years.

Similar to results in adults (29) and children (30), we showed that the most significant drop in heart rate (increase in mean NN) occurs between sleep onset and SWS1 and continues to drop only in SWS cycles as time asleep progresses. Unlike Baharav et al. who in 10 children and adolescents showed no difference in heart rate between sleep stages (31), we showed that heart rate (mean NN) was significantly different between some SWS periods and REM sleep periods across the night but that all sleep stage cycles were not the same in uninterrupted sleep. For example the first SWS (SWS1) cycle reported a similar mean NN to all REM cycles, with the lowest mean NN and highest heart rate recorded during REM3. However, mean NN was significant higher (heart rate lower) in SWS2 and SWS3 compared to SWS1 and all REM periods. Of significance is the dramatic difference in cardiac cycle length (7 beats per minute) between SWS3 and REM3 which occurred approximately an hour apart in most children. What drives these changes in heart rate is difficult to ascertain from our data, however we suspect this may be evidence of an ultradian biorhythm in heart rate (32) that is reported to be autonomic in origin.

SDNN, considered a composite of all factors that contribute to HRV (33), remained similar to pre-sleep levels in all SWS cycles and REM3 but significantly increased by REM2 and REM1. The pre-sleep measurements were extracted from the signal 3 min prior to the sleep onset time, which was determined using the EEG signals, and the Rechtschaffen and Kales rules. Heart rate was highest and mean NN was lowest during pre-sleep yet SDNN, a marker of autonomic modulation, was at its lowest. Most children had been resting supine in their bed for more than 10 min before they fell asleep. It is proposed that variations in SDNN during short resting recordings reflects parasympathetically-mediated respiratory sinus arrhythmia especially during slow paced breathing protocol (33). This relaxation time and more paced breathing may account for the reduction in SDNN prior to sleep onset. However, one would think that heart rate would also decrease in a similar manner if this was indeed the case. Our data suggests whatever parameter that increases SDNN “reactivates” in the early hours of the morning before wake. As SDNN during REM3 and SWS3 were almost identical, it is unlikely that changes in SDNN during sleep are driven by the same mechanisms associated with sleep staging, e.g., ultradian fluctuations as the case of mean NN.

SDNN is best measured over 24 h recording as they provide data on cardiac response to environmental stimuli. However, using SDNN in short segments at times where we know there are physiological changes that are not related to movement and mental stress, but are associated with “autonomic change” we might better understand the base level of the autonomic input (22). The increase in SDNN in the REM periods just before waking may reflect the increase in heart rate (reduced mean NN) however it does not explain the increase in heart rate (reduced mean NN) during REM3 where SDNN is similar to the subsequent SWS3 and where the mean NN is the highest (lowest heart rate). Irregular respiratory rate is more prominent during REM sleep compared to SWS where respiratory rate is stable and respiratory rate is slower. Irregular respiratory rate may account for the increase in REM periods, but doesn't explain the similar SDNN in REM3, which suggests respiratory stability was conserved.

RMSSD is a measure of the beat-to-beat difference in heart rate and both rMSSD and HF are considered indicators of vagal input in HRV (22) and increases in either variable indicate increase parasympathetic tone to the cardiac tissue. Both measures were lowest before sleep onset but increased significantly during SWS1. As sleep progressed, rMSSD values remained constant regardless of stage and time of night except during REM3 where it reduced but not to pre-sleep levels. HF remained at the same level across the night. If rMSSD and/or HF reflect the activity of the vagal efferents then our data suggests that there is a large increase in vagal activity once sleep onsets, not before. Conversely, given the large reduction between pre-sleep SDNN and SDNN in REM periods the change in rMSSD/HF may reflect a disengagement of sympathetic efferents to cardiac tissue, which may reveal the optimal resting vagal input that remains stable across sleep. Our results also imply that in children and adolescents the vagal input is less effected by ultradian cycling and possibly even independent of the circadian influence. The significant reduction in rMSSD we observed during REM3 may be an indicator of the influence of circadian oscillator however the drop in rMSSD did not continue in subsequent cycles, which is what we would have expected to occur if the vagal measurements were influenced by the circadian pacemaker coming into the early hours of the morning. The drop in rMSSD in REM3 may result from a reduction in vagal cardiac innervation (or increase in sympathetic innervation) and is consistent with an increased heart rate and decrease mean NN at that same time, which is what occurred. Whether this a part of a centrally mediated program of the circadian cycle or an anomaly of the results needs to be further investigated.

The physiological implications of LF have been proposed to reflect baroreflex, and vascular compliance during rest (6, 8, 34) and not cardiac sympathetic innervation (35) as previously suggested (36). Although we have not measured vascular resistance directly during sleep we have measured sleep stages and the position of the cycle across the dark phase of the circadian cycle, which are documented times related to different vascular states (37) (SWS1 and 2, which occur early in the night and are associated with vasodilation, and REM1 and 2, which are stages that occur closer to wake time and are associated with significant vasoconstriction). Our LF results may demonstrate the activation of vasomotor pathways to the peripheral vessels and in particular cutaneous vasculature that may be required to initiate behaviors specifically associated with sleep (10). Inhibition of sympathetic vascular innervation to cutaneous vessels is a feature of vasodilation and heat loss (38) which is evident during sleep onset and during SWS (39) and our results of low LF and LF:HF in the first SWS cycle may reflect this vasodilation. LF was increased during pre-sleep and decreased by half in SWS1. LF stayed low for the first half of the night then continued to increase incrementally as the night progressed, also independent of sleep stage. Consistent with our findings, Trinder et al., showed an abrupt drop in blood pressure during the first SWS period in healthy young adults (40). The increase in LF across the night may be an indicator of gradual vasoconstriction of cutaneous vasculature occurring after a large initial drop in total peripheral resistance required to activate mechanisms that onset deep sleep. The gradual reduction in peripheral vascular compliance would increase heat conservation and preserve core temperature as sleep progresses and ambient temperature drops, regardless of the sleep stage classification. With the exception of pre-sleep, SDNN followed the same pattern as LF during sleep and supports the correlation reported by others between the two variables (33). Furthermore, the significant increase in LF, LF:HF and SDNN in the REM1 and REM2 coincides with previously reported increases in heat production in adult males (41) and REM sleep has been associated with increased vasoconstriction (41). Heart rate increased and mean NN decreased to almost pre-sleep levels in these two cycles, however the highest HF (a potential indicator of parasympathetic drive to cardiac tissue) was reported to occur in REM2 and REM1 suggesting the increase in heart rate was not mediated by changes in vagal innervation. Inhibition to neurons in the rostral raphe and the RVLM brain stem regions abolishes vasoconstriction to cutaneous vessels during mild cooling (10). Both the raphe and the RVLM regions directly control premotor pathways to cardiac tissue and the baroreflex vasomotor control suggesting a link between, cardiac function, peripheral vascular control, and thermoregulation (10). Perhaps the changes in LF, SDNN, and LF:HF we observed during the REM periods is evidence of the interrelationship, vascular compliance, cardiac function, and thermoregulation.

Silvani et al. points out that “the integration between central and baroreflex drives, which produces the cardiovascular autonomic outflow, not only differs between wakefulness and NREM, but also between the two sleep states.” They further speculate that inconsistences in the relationship between heart rate, arterial blood pressure, and cardiac output during sleep arise from changes in vascular compliance (42) suggesting a possible role for thermoregulation. Our results support this premise in that, using HRV variables, one can have the same heart rate/meanNN and very different underlying “autonomic cardiovascular outflow” (SDNN, LF, HF, rMSSD) throughout the night and that these autonomic indicators can be different within the same sleep stage. Given the interrelationship between cardiac function and vascular motor control, and that elements of HRV such as LF, SDNN, and LF:HF during sleep in this study may measure peripheral vasomotor control, sleep maybe an ideal time to investigate changes in the integrated physiology, e.g., cardiac activity, vasomotor function, and thermoregulation.

We surmise if LF is a measure of vascular compliance then our results fit the current model of sleep where LF picks up vasoconstriction in REM sleep closer to waking and vasodilation when sleep onsets. Using “uninterrupted” sleep segments across the night during different sleep stages we may better understand vascular pathology, particularly in the pediatric population (43, 44).

### Interpreting the Inconsistencies

Our data points to possible inconsistencies in the interpretation of HRV results during sleep in some previous studies. We demonstrated that the SDNN prior to sleep onset was the lowest compared to all the other times recorded, and that this was not the case with all the other HRV variables. If SDNN is a composite of SNS and PNS activity (or potentially any other mediator of cardiac change) why are rMSSD and HF still low and LF still high at this time? Our results suggest that whatever is driving changes in SDNN becomes quiescent before the participants entered sleep and what drives other HRV variables activate once sleep has onset.

Also, in REM1 and REM2, SDNN, LF, and LF:HF are significantly increased and HF is the same compared to SWS1 (SDNN, LF, and LF:HF were at their lowest point in the night in SWS1) yet the heart rates/meanNN were the same, suggesting whatever caused the higher heart rates (lower meanNN) in SWS1 and REM periods come from different mechanisms. Furthermore, why are SDNN, rMSSD, LF and HF similar during SWS1 and SWS2&3 yet the heart rates/mean NN significantly different? We postulate two hypotheses. First that the increased heart rate during this first SWS period may reflect the increase in concentration of circulating cardiovascular modifiers [humoral (melatonin/cortisol) and catecholamine], which are high in the acrophase of the circadian cycle (45–47) acting directly on cardiac tissue. As time in sleep progresses these modifiers dissipate and the heart rate reduces revealing the autonomic input.

Or second, our results in the first SWS cycle may reflect the effect of increased body temperature on cardiac tissue conductance (39, 48) independent of neuronal input to the heart itself as body temperature is at its peak just prior to sleep onset (acrophase) and is dropping in the first sleep cycle but still high in the early hours of sleep (12). Cardiac tissue cooling in rabbits has demonstrated an increase in spontaneous sinus cycle length, suggesting hotter core temperature alone could account for the higher heart rate in SWS1 (49). Hence the lengthening of the cardiac cycle may occur as a bi-product of somatic cooling. While the increase in heart rate and decrease in mean NN during REM periods may result from an increase in cutaneous vasoconstriction that increases vascular resistance and activation of facultative heating mechanisms that increase body temperature preparing the brain and organs for arousal from sleep (10).

The inconsistences in our results may be an example of “baroreflex resetting” proposed by Dampney as a capacity of baroreceptors to alter their activation to mediate a proposed behavior, in this case sleep onset and sleep maintenance (50). Dampney also highlights that cutaneous vasomotor activation is less dependent on baroreflex input compared to sympathetic vasomotor outflows to other vascular beds and hence the changes in LF we describe may be mediated independent of the baroreflex and evidence of centrally activated pathways associated with thermoregulation.

### Theoretical Application

It has been proposed that SWS is an ideal time to measure HRV because of its relative “stability,” with less movement and regular respiratory patterns (51), however, our data suggests that not all SWS periods across the night are the same. The differences between the SWS cycles requires more investigation to elucidate the parameters (exogenous e.g., catecholamine levels, vascular compliance, etc.) that modulate the cardiac changes. Our results have implication for studies that measure HRV between groups during the day. Both circadian and ultradian rhythms affect daytime body temperature and heart rate, hence data collection between two different groups (e.g., exercise data) need to be collected at the same time of day to reduce this confounding effect.

If LF is an indicator of vascular compliance it may explain the higher incidents of heart attack (52) and stroke (53) reported in the mornings in adults. Increased vascular resistance requires a stronger inotropic output and may put more pressure on an already compromised system, hence increasing the probability of cardiac incidences in the morning. Assessment of pre-wake REM sleep SDNN, LF, and LF:HF may be prognostic of possible cardiovascular disease vulnerability.

Our data may also explain the discrepancy between automating sleep software that use HRV to classify sleep staging (54, 55). We showed differences in HRV between cycles that would need to be applied to an algorithm designed to distinguish sleep stages when only using HRV changes to define stage differences, such as in the case of basic smart phone applications and new sleep home monitoring systems.

Many HRV studies in sleeping children assess the relationship between age and heart rate variability to measure the “maturation of the autonomic capacity” (56–58) or to determine HRV differences in children with disorders affecting cardiac function such as sleep disordered breathing (59). These studies all yield different information as the protocol of when to measure HRV at night are not consistent and most amalgamate and average data from like stages across the night. The strength of this study is the large sample number, the consistent and separated interrogation and comparison of data at similar time points across the night and wide age range which increases the likelihood that the findings in this study are robust. Our results also concur with the suggestion by Scholz et al. that using the average heart rate over short and long periods (24 h recordings) of time underestimates autonomic activation (60). Furthermore it highlights the value in independently assessing HRV changes using data from uninterrupted sleep in different cycle across the night. The differences in HRV variables we have demonstrated across the night may all have different underlying parallel pathways that equally effect the way the heart pumps blood around the body. More discrete analysis of “sleep” HRV may give us better understand of the role these cardiovascular mediators play in cardiac physiology and pathology.

One of the challenges of assessing HRV during sleep is to obtain consistent, artifact free signal over a long period of time in a specific sleep stage. REM makes up ~25% of sleep across the night, with short periods occurring at the beginning of sleep and bouts of REM increasing as sleep progresses toward the morning. To maximize the number of data points and facilitate data analysis we addressed this limitation by labeling the stages and cycles as described.

## Conclusion

Our study has shown that cardiac function during sleep is significantly modulated by both ultradian and circadian parameters. Importantly, our findings support the hypothesis that LF and LF:HF measure vascular compliance via baroreceptor, and/or centrally controlled factors (e.g., temperature control) contingent upon ultradian and circadian rhythms. Findings from studies that have shown differences in LF values should be reconsidered in the light of the current results. There is evidence that some disease states that also affect cardiovascular function are associated with changes in circadian and ultradian cycling. Hence, differences in HRV variables measured throughout the night during different sleep stages (REM and SWS) may indicate different underlying pathology. By better understanding their contribution during sleep in healthy children it may give us better insight into the origin and treatment of these diseases, many of which begin in childhood.

## Data Availability Statement

The datasets generated for this study will not be made publicly available. We have not got consent from parents to release raw data to a third party. Requests to access the datasets should be directed to Anna Kontos, anna.kontos@adelaide.edu.au.

## Ethics Statement

The studies involving human participants were approved by the Human Research Ethic Committees of Women's and Children's Hospital Network, University of South of Australia, and University of Adelaide. Written informed consent to participate in this study was provided by the participants' legal guardian/next of kin.

## Author Contributions

AK and MB: study design. AK, MB, MK, DC-N, and YP: data collection. AK, MB, and KL: data analysis. AK and MB: interpretation of results. AK, MB, DK, KL, JM, MK, DC-N, and YP: preparation of manuscript.

### Conflict of Interest

The authors declare that the research was conducted in the absence of any commercial or financial relationships that could be construed as a potential conflict of interest.
